# Standardizing MR defecography referral and reporting: a multidisciplinary Delphi consensus

**DOI:** 10.1007/s11547-026-02279-w

**Published:** 2026-09-21

**Authors:** Anna Colarieti, Ugo Grossi, Marco Oderda, Edda Battaglia, Pietro Biondetti, Giampiero Capobianco, Elsa Beltrami, Silvia Cornaglia, Elisabetta Costantini, Marco Estienne, Mara Falco, Raffaella Ferrando, Nicoletta Gandolfo, Gianfranco Lamberti, Daniela Lo Sasso, Francesca Maccioni, Gabriele Naldini, Arcangelo Picciariello, Fausto Pittarello, Marco Ranucci, Alfonso Reginelli, Mara Scabini, Daria Schettini, Tommaso Simoncini, Angelo Stuto, Marco Torella, Vittorio Piloni, Paolo Tonello

**Affiliations:** 1 Università Degli Studi del Piemonte Orientale Amedeo Avogadro, Dipartimento Di Medicina Traslazionale, Novara, Italy; 2 Coloproctology Unit, Humanitas Gavazzeni E Castelli, Bergamo, Italy; 3 Università Degli Studi Di Torino, Città Della Salute E Della Scienza Di Torino - Ospedale Molinette, SC Urologia, Turin, Italy; 4 Dipartimento Di Gastroenterologia ASL TO4, Ospedale Di Chivasso, Turin, Italy; 5 Dipartimento Di Radiologia, Policlinico Abano Terme, Padua, Italy; 6 Director of Complex Unit of Gynecology and Obstetrics, Università Di Sassari, Sassari, Italy; 7 Pelvic Floor Disease Center, Ospedale Koelliker, Turin, Italy; 8 Direttore Clinica Urologica ad Indirizzo Andrologico e Uroginecologico AOU, Terni, Italy; 9 Vivolab Istituto Radiologico Diagnostico, Chiavari, Genoa, Italy; 10 Director of Department of Radiology, Ospedale Koelliker, Turin, Italy; 11 Department of Radiology, IRCCS Policlinico San Donato, Milan, Italy; 12 Dipartimento di Radiologia, Ospedale Villa Scassi, Genoa, Italy; 13 Department of Medicine and Surgery, University of Parma, Parma, Italy; 14 Department of Radiology, Casa di Cura Clinica Candela, Palermo, Italy; 15 Department of Radiology, Università Sapienza, P. Umberto I, Rome, Italy; 16 Division of Colon and Rectal Surgery Humanitas Hospital, Milan, Italy; 17 Assistant Professor of Surgery, Department of Experimental Medicine, University of Salento, Lecce, Italy; 18 UOC Radiologia, Piove Di Sacco, Padua, Italy; 19 Department of Cardiovascular Anesthesia and Intensive Care, IRCCS Policlinico San Donato, San Donato Milanese, Italy; 20 Dipartimento Di Medicina Di Precisione, Università Della Campania Luigi Vanvitelli, Naples, Italy; 21 Department of Radiology, ASST Pavia, Pavia, Italy; 22 Dipartimento Immagini, ASL3 Liguria, Genoa, Italy; 23 Dipartimento di Medicina Clinica e Sperimentale, Divisione Ginecologia e Ostetricia, Università Degli Studi di Pisa, Pisa, Italy; 24 Chair ColoProctology and Pelvic Floor Unit, IRCCS San Donato, Milan, Italy; 25 Dipartimento di Ginecologia, Università della Campania Luigi Vanvitelli, Naples, Italy; 26 Diagnostica Marche, Ancona, Italy

**Keywords:** Delphi consensus, Magnetic resonance defecography, Multidisciplinary collaboration, Pelvic floor disorders, Reporting template

## Abstract

**Purpose:**

Dynamic magnetic resonance defecography (MRD) is a key imaging modality for comprehensive evaluation of pelvic floor dysfunction, enabling detailed multiplanar anatomical and functional assessment without ionizing radiation. However, despite its established clinical role, MRD reporting is heterogeneous among centers, hindering standardized interpretation, multidisciplinary communication, and comparison across studies. This modified Delphi consensus study aimed to develop a standardized referral dataset and structured reporting framework for MRD.

**Methods:**

A multidisciplinary panel of 20 Italian experts (radiologists, colorectal surgeons, gynecologists, urologists, gastroenterologists, physiatrists, and a methodologist) conducted a three-round Delphi process to develop consensus-based templates for MRD referral and reporting. Consensus was set at ≥75% agreement. A total of 36 items were evaluated, covering technical parameters, anatomical/functional findings, and reporting format.

**Results:**

Consensus was achieved for 21 items (58%), including technical parameters, standardized measurements (anorectal angle, pubococcygeal line), and clinically relevant descriptors (prolapse, dyssynergia, rectocele, rectal inertia). A structured referral template was also developed to improve information transfer from clinicians to radiologists. Both tools were unanimously endorsed.

**Conclusions:**

This modified Delphi consensus study provides a multidisciplinary framework for standardized MRD referral and reporting. The proposed reporting template may facilitate greater consistency in image interpretation, reporting terminology, and multidisciplinary communication. These recommendations may serve as a foundation for the harmonization of MRD practice and future validation studies in pelvic floor imaging.

## Introduction

Dynamic magnetic resonance defecography (MRD) is widely recognized as a key imaging modality for the comprehensive evaluation of pelvic floor disorders (PFD), which are highly prevalent among women and represent a major public health concern [1]. The burden of PFD is expected to rise further due to demographic aging and the growing prevalence of risk factors such as obesity, multiparity, and prior pelvic surgery.

PFD-related symptoms may arise from structural pelvic floor abnormalities, functional disorders such as dyssynergic defecation (paradoxical contraction or inadequate pelvic floor relaxation during evacuation), or a combination of both within the same patient [2]. Accurate clinical, radiological, and physiological assessment is therefore essential to appropriately phenotype patients and guide management.

In this context, MRD provides multiplanar, high–soft tissue–contrast, dynamic assessment of pelvic anatomy and function without exposure to ionizing radiation, making it particularly suited for the evaluation of functional pelvic floor abnormalities [3, 4]. MRD has demonstrated value in the evaluation of posterior compartment disorders—such as rectocele, enterocele, sigmoidocele, and rectal intussusception—as well as in complex, multicompartmental PFD, where clinical examination alone is frequently insufficient or misleading [5, 6].

Several studies have shown that clinical examination may underestimate, misclassify or incompletely characterize PFD, with potential consequences on treatment selection and surgical outcomes [7, 8]. In this setting, MRD has been shown not only to improve diagnostic accuracy but also to influence clinical decision-making, including surgical planning, particularly in patients with posterior compartment prolapse or obstructed defecation [8]. Consequently, MRD is increasingly incorporated into multidisciplinary diagnostic pathways involving radiologists, colorectal surgeons, gynecologists, urologists, and gastroenterologists.

Despite its established diagnostic value, MRD reporting remains highly heterogeneous across institutions and operators. International multisociety efforts have recently proposed standardized MR defecography lexicons and reporting templates [9]. However, these initiatives were not developed through a national, multidisciplinary Delphi process aimed at defining both minimum referral information and a feasible core reporting dataset tailored for routine implementation across heterogeneous centers. As a result, reporting practices still vary in structure, terminology, choice of reference lines, inclusion of dynamic phases, and emphasis on clinically relevant findings. This heterogeneity hampers effective communication between radiologists and referring clinicians, reduces interobserver reproducibility, and limits the comparability of data across centers, thereby undermining both patient management and multicenter research efforts [8, 10–12].

Structured reporting has been widely adopted in multiple areas of radiology as a tool to improve clarity, completeness, and consistency of radiologic communication, while supporting multidisciplinary collaboration and data standardization [13–15]. In pelvic floor imaging, however, no nationally shared, multidisciplinary consensus exists regarding what information should be systematically included in MRD referrals and reports, nor how these findings should be presented in a clinically oriented and reproducible manner. Importantly, MRD is not a purely technical radiological examination but a clinically driven, question-oriented investigation, whose diagnostic value depends heavily on the quality of the referral information and on the report’s ability to address specific clinical concerns. This necessitates a shared language and agreed-upon priorities between imaging specialists and referring clinicians.

To address this unmet need, we conducted a multidisciplinary Delphi consensus involving Italian experts with established experience in PFD. The objectives were to define, through structured expert consensus, (1) the minimum clinical information required when requesting MRD, and (2) a core set of technical and anatomical-functional items that should be consistently included in MRD reports to improve clarity, reproducibility, and clinical utility. The goal of this work is to provide a consensus-based, structured template for MRD reports that can facilitate interdisciplinary communication and lay the groundwork for future multicenter validation studies. **Summary Statement** This modified Delphi consensus developed the first nationally shared, multispecialty template for referral and reporting in magnetic resonance defecography, aiming to improve clarity, standardization, and interdisciplinary communication in the evaluation of pelvic floor disorders.

## Methods

### Study design

This study was designed as a methodological, consensus-based investigation using the Delphi technique [16]. This modified Delphi method is a structured process aimed at achieving agreement among experts through iterative rounds of anonymous rating, feedback, and refinement and is particularly suited for areas where empirical evidence is heterogeneous or where standardization is lacking. This study did not involve human participants, patient data, or imaging review. According to institutional and national regulations, ethics committee approval and informed consent were not required.

### Panel composition and expert selection

The Delphi process involved two sequential expert panels.

An initial steering panel of five opinion leaders in PFD convened to define the scope and draft preliminary statements. Panel members were selected based on: (1) documented clinical experience in PFD for at least 5 years; (2) active involvement in MR defecography interpretation or referral; and (3) peer-reviewed publications in pelvic floor imaging or management.

This steering panel comprised three colorectal surgeons (E.B., S.C., P.T.) and two radiologists (M.F., V.P.).

Subsequently, a second multidisciplinary panel of 20 Italian experts was assembled using purposive sampling to ensure balanced representation of all specialties involved in pelvic floor care. The final panel included 8 radiologists, 4 gynecologists, 3 colorectal surgeons, 2 urologists, 1 gastroenterologist, 1 physiatrist, and 1 methodologist. All panelists had documented expertise through clinical practice, scientific publications, and leadership roles in pelvic floor units.

### Literature review and statement development

Prior to the Delphi rounds, the steering panel performed a focused narrative review of the English-language literature using PubMed, with the search terms *“MR defecography”* AND *“pelvic floor dysfunction”*. This aimed to summarize current evidence regarding MRD indications, technical execution, measurement systems, reporting practices, and clinical impact.

Based on the literature review and expert discussion, two groups of statements were developed:

1. Statements addressing minimum clinical information required for MRD referral.
2. Statements addressing technical, anatomical, and functional items to be included in a structured MRD report.

All statements were reviewed and refined with methodological support from an experienced methodologist (M.R.) to ensure clarity, neutrality, and consistency with Delphi methodology.

### Delphi process and consensus definition

**Fig. 1:**
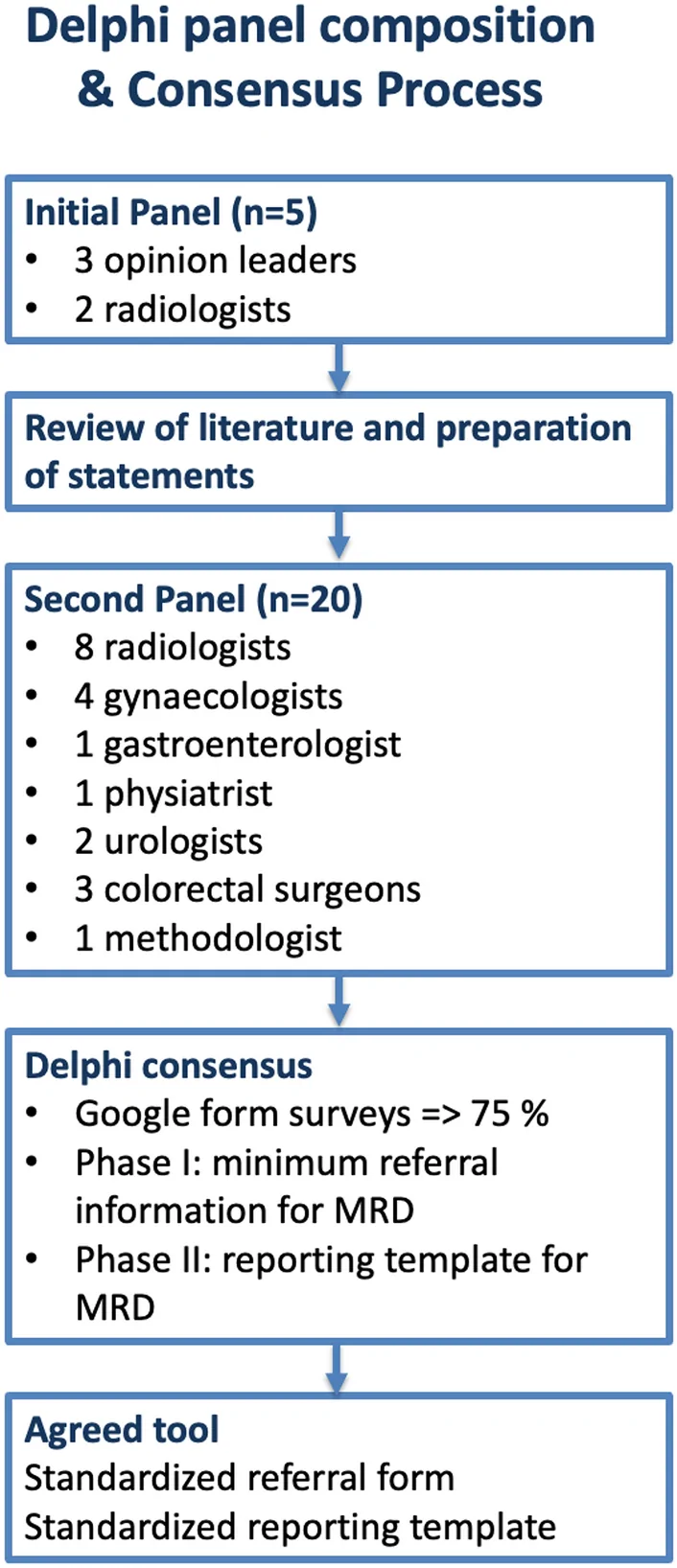
Delphi panel composition and workflow. MRD: magnetic resonance defecography

The consensus process consisted of three iterative Delphi rounds conducted electronically (Google Forms). Statements were rated using a four-point Likert scale: *strongly agree*, *partially agree*, *partially disagree*, *strongly disagree* (Fig. 1). Consensus was predefined as ≥75% of respondents selecting *strongly agree* or *partially agree*. Following each Delphi round, statements that failed to achieve the predefined consensus threshold were systematically reviewed by the five-member steering committee. Revisions were informed by both the quantitative voting results and the anonymized qualitative comments provided by panelists. Proposed modifications were aimed at improving clarity, reducing ambiguity, and addressing concerns raised during the evaluation process. The revised statements were subsequently discussed during plenary meetings and finalized by the steering committee before redistribution to the entire expert panel for anonymous reassessment in the subsequent round. Although discussion was permitted between rounds, all consensus decisions were ultimately based on anonymous voting by the full panel, consistent with the principles of a modified Delphi methodology.

### Outcomes of the Delphi process

The Delphi consensus occurred in two phases aimed to produce two complementary tools:

1. Phase I: The expert panel addressed the lack of standardization in MRD referral practices, which contributes to heterogeneity in acquisition protocols and challenges in image interpretation. A minimum referral information dataset to standardize clinical information provided to radiologists when requesting MRD was developed.
2. Phase II: A standardized structured reporting template for MRD was developed, defining a core dataset of technical and anatomical-functional elements to be systematically reported.

## Results

### Delphi consensus process

#### Phase I–Delphi consensus on minimum referral information

An initial draft comprising 11 statements was developed and refined through three iterative Delphi rounds. Anonymous voting was complemented by plenary e-meetings to discuss and resolve borderline items.

Consensus was achieved for all 11 statements. This led to the development of a minimum referral information dataset, which was unanimously endorsed by the panel (Table 1).

**Table 1:** Minimum referral information dataset for MRD

Referral item	Mandatory
Patient demographics and contact information (name, surname, sex, date of birth, and contact details)	✓
Referring physician identity and contact	✓
Specific diagnostic question	✓
Parity (obstetric history)	✓
Prior pelvic surgery	✓
Fecal incontinence	✓
Urinary incontinence	✓
Obstructed defecation	✓
Obstructed voiding	✓
Chronic pelvic pain	✓
Pelvic organ prolapses (if possible, by compartment)	✓

The final referral template mandates the inclusion of key demographic, clinical, and functional history elements considered essential to guide MRD protocol selection and ensure accurate and clinically meaningful interpretation.

#### Phase II–Delphi consensus on structured MRD reporting

Based on a narrative literature review and feedback collected during the initial panel meeting, 36 candidate statements were generated. These statements were evaluated over three rounds of anonymous voting, with iterative revisions guided by qualitative feedback. Remote plenary sessions were subsequently held to discuss borderline items and promote convergence.

Overall, consensus was achieved for 21 out of 36 statements (58.3%), while 15 statements (41.7%) were rejected (Table 2).

**Table 2:** Delphi consensus summary

Delphi item category	Total statements (n=36)	Accepted (%) (n=21, 58%)	Rejected (%) (n=15, 42%)
Technical (radiologists only)	11	11 (100)	0 (0)
Anatomical/functional (all panelists)	25	10 (40.0)	15 (60.0)

The accepted statements (Table 3) included both technical parameters (11/11; 100%) and anatomical/functional parameters (10/25; 40%). Collectively, these items defined a core dataset intended to improve interobserver reproducibility, reporting completeness, and clinical applicability across institutions.

- Technical consensus items focused on standardizing acquisition and measurement details, including reporting of magnet characteristics, patient preparation and positioning, contrast administration, imaging phases, and key quantitative measurements such as the anorectal angle and pelvic reference lines.
- Anatomical and functional consensus items addressed the systematic evaluation of PFD across compartments, functional abnormalities, and the inclusion of a structured final impression aligned with the clinical referral question.

**Table 3:** Statement-derived items to enter a structured MRD report

Category	Item	Agreement (%)
Technical	Magnet type (open/closed)	84.6
	Field strength	92.3
	Bowel preparation and type	76.9
	Patient position during defecation phase	77.0
	Acquisition of static/dynamic phases	100
	Type and amount of contrast agent	76.9
	Contrast administration site(s) (rectum, vagina, bladder, bowel)	76.9
	Imaging planes acquired	76.9
	Anorectal angle measured at rest, squeeze, strain, evacuation	84.6
	H-line, M-line, O-line relative to pubococcygeal line	76.9
	Any contrast leakage during filling phase	100
Anatomical/functional	Puborectalis dyssynergia	85.0
	Prolapse(s) in the anterior/middle/posterior compartments	95.0
	Rectocele	95.0
	Intussusception (recto-rectal/recto-anal)	95.0
	Enterocele / sigmoidocele	95.0
	Rectal inertia (Impaired rectal evacuation)	95.0
	Urethro-vesical angle modifications	100
	Structural visceral/muscular alterations (e.g., levator avulsion)	100
	Structured final impression addressing the clinical question	85.0
	Suggestion for any further diagnostic work-up	80.0

The 15 rejected statements (Table 4) predominantly concerned items considered technically burdensome, insufficiently standardized in the literature, or beyond the radiologist’s diagnostic remit. These included additional pelvic floor measurements (e.g., levator hiatus dimensions), post-evacuation imaging protocols, reporting of hardware specifications, bibliographic referencing of measurement systems, and radiologist-driven therapeutic recommendations.

**Table 4:** Statements not accepted after Delphi rounds

Statement	Agreement (%)
Report magnet position (i.e., horizontal or vertical)	69.2
Include post-evacuation images after toilet use	69.2
Report diameters/area of levator hiatus	69.3
Measure rectal diameters at maximum filling and post-evacuation	69.3
Describe number/type of expulsive acts	69.2
Report site and quantity of residual contrast	69.3
Specify the coil type (body vs. phased array)	46.2
Report the sequence (whether static or dynamic was acquired first)	53.9
Timing of the static phase acquisition relative to contrast administration	46.2
Include bibliographic footnotes for measurement systems and anatomical landmarks	61.6
Report diameters of the anal canal at rest and during evacuation	53.9
Include the levator plate angle and infrapubic horizontal line	61.6
Report the percentage/fraction of contrast expelled	61.6
Radiologist should suggest a medical treatment	20.0
Radiologist should suggest a surgical treatment	20.0

Although several statements approached the predefined consensus threshold (e.g., levator hiatus measurements, 69.3%), they were excluded to maintain feasibility, reduce reporting complexity, and limit interobserver variability.

At the conclusion of the Delphi process, the panel unanimously endorsed both standardized tools. A representative example of the final consensus-derived structured MR defecography report is provided below to facilitate understanding and practical implementation of the proposed framework (Table 5).

**Table 5:** Structured MRD Reporting Template

Section	Item	Details/fields
Patient and referral information	Patient demographics and contact information	Name, surname, sex, date of birth, contact details
	Referring physician	Name and contact information
Clinical history	Parity	Obstetric history
	Prior pelvic surgery	Yes / No (specify)
	Symptoms	Fecal incontinence (Yes / No) Urinary incontinence (Yes / No) Obstructed defecation (Yes / No) Obstructed voiding (Yes / No) Chronic pelvic pain (Yes / No)
	Pelvic organs prolapse	Known or suspected prolapse (specify compartment)
Technical parameters	Magnet type	Open / Closed
	Field strength	___ Tesla
	Bowel preparation	Yes / No (specify type)
	Patient position during defecation phase	Supine / Sitting / Other (specify)
	Static phases acquired	Yes / No (specify planes)
	Dynamic phases acquired	Yes / No (Rest / Squeeze / Strain / Evacuation)
	Contrast agent type	___
	Contrast agent amount	___ mL
	Contrast administration sites	Rectum / Vagina / Bladder / Bowel
	Imaging planes acquired	Sagittal / Axial / Coronal
	Anorectal angle	Rest: ___°; Squeeze: ___°; Strain: ___°; Evacuation: ___°
	H-line	___ mm
	M-line	___ mm
	O-line	___ mm
	Contrast leakage during filling phase	Yes / No
Anatomical and functional findings	Compartmental prolapse	None / Present (Anterior, Middle, Posterior compartment)
	Puborectalis dyssynergia	Absent / Present
	Rectocele	Absent / Present (specify size)
	Intussusception	Absent / Present (Recto-rectal / Recto-anal)
	Enterocele / Sigmoidocele	Absent / Present
	Rectal inertia	Absent / Present
	Urethro-vesical angle modifications	Absent / Present
	Levator avulsion / muscular alterations	Absent / Present
Structured final impression	Summary of findings	Key findings relevant to the clinical question
	Compartment(s) involved	___
	Functional alterations	___

## Discussion

This Delphi consensus represents the first multidisciplinary initiative in Italy aimed to standardize both the referral process and the reporting of MRD in patients with PFD. The two tools developed are designed to improve communication between referring physicians and radiologists, reduce interobserver variability, and promote a shared language across specialties [17].

A key strength of this initiative lies in its multispecialty integration. PFD are inherently complex, often involving more than one anatomical compartment, and their optimal management requires the coordinated input of colorectal surgeons, gynecologists, urologists, radiologists, gastroenterologists, and physiatrists. The consensus process confirmed that radiological reporting cannot be conceived in isolation but must be tailored to the specific needs of referring clinicians. The explicit rejection of radiologist-driven therapeutic recommendations (20% agreement) underscores the importance of preserving professional boundaries while fostering a constructive diagnostic–clinical dialog.

From a technical and practical perspective, the structured template emphasizes feasibility and reproducibility. Accepted items prioritized parameters widely recognized in the literature and feasible in routine practice, such as the anorectal angle, H/M/O lines, and prolapse grading across compartments [12]. Conversely, technically demanding or poorly standardized parameters (e.g., levator hiatus diameters, post-evacuation measurements) did not reach consensus. This pragmatic balance ensures that the proposed dataset can be consistently applied across centers without introducing excessive variability or extending reporting time beyond what is sustainable in daily clinical activity.

The adoption of structured reporting for MRD confers multiple methodological, clinical, and research-related advantages. By enforcing standardized terminology, predefined reporting fields, and systematic documentation of both technical and interpretative parameters, structured MRD reporting improves report completeness, internal consistency, and clarity. This structured approach may reduce interpretative ambiguity and interobserver variability, which are well-recognized limitations of narrative reporting, particularly in complex functional imaging examinations [15, 18].

Importantly, the value of structured reporting extends beyond reporting standardization. The systematic collection of quantitative measurements, functional parameters, and clinically relevant descriptors generates high-quality, interoperable datasets that can facilitate multicenter research, quality assurance initiatives, and longitudinal outcome studies. Moreover, the adoption of standardized reporting frameworks represents a prerequisite for the development of advanced data-driven approaches, including radiomics and artificial intelligence applications, by enabling reproducible extraction and comparison of imaging-derived information across institutions. In this context, structured MRD reporting may contribute to the future identification of imaging biomarkers and support the development of more objective and reproducible assessment strategies for pelvic floor disorders.

From a clinical perspective, structured reporting facilitates more effective communication among radiologists, surgeons, gastroenterologists, and pelvic floor specialists by ensuring that imaging findings are explicitly linked to the clinical indications and diagnostic questions that prompted the examination. This alignment enhances the clinical relevance of MRD reports and supports more informed multidisciplinary decision-making, with potential downstream benefits for patient management and treatment planning [19].

Beyond individual patient care, standardized MRD reporting establishes a reproducible and harmonized framework for data collection across institutions. This uniformity is essential for multicenter clinical research, enabling reliable aggregation and comparison of imaging data, reducing heterogeneity in outcome measures, and improving the robustness of observational studies and clinical trials. Structured reports also provide a consistent basis for quality assurance, auditing, and performance benchmarking, supporting continuous improvement in imaging practice [14].

Nonetheless, some limitations must be acknowledged: structured templates may reduce flexibility, potentially omitting rare or atypical findings not included in the dataset. For instance, the assessment of megarectum—often defined by transverse rectal diameter in the mid rectum—was not retained in our consensus, despite previous work highlighting its potential clinical relevance [20]. The exclusion of borderline items, while necessary for feasibility, may risk overlooking emerging but not yet validated parameters; and the successful implementation of structured reporting requires dedicated training and a cultural shift among radiologists and clinicians alike.

Comparable initiatives have been undertaken in other fields of imaging. For example, structured MR reporting templates have been developed for anal fistula [11], endometriosis [15], multiple sclerosis [21], and malignant diseases [22–24], with evidence suggesting improvements in report quality, reproducibility, and clinical usability.

In the field of PFD, international efforts have been undertaken to standardize reporting across different modalities. For example, Paquette et al. [8] described a consensus-based structured template for fluoroscopic (barium) defecography, while Gurland et al. [7] proposed standardized terminology and interpretative criteria for MRD with multispecialty endorsement. More recently, Piloni et al. [25] developed an Italian structured reporting format for MRD including nine descriptors, based on literature review and expert opinion. While these initiatives represent important contributions, the former primarily addressed fluoroscopic imaging, the second focused on MRD terminology and interpretation without a formal reporting dataset, and the latter was mainly radiology-driven without a consensus methodology. In addition, Revels et al. [9] synthesized these consensus efforts and provided practical reporting templates and lexicon recommendations, mainly emphasizing objective measurements and compartment-based assessment. Our Delphi process expands on these initiatives by systematically integrating multiple specialties and by producing two complementary tools (a referral dataset and a reporting template) formally endorsed through consensus.

Building on these results, the next step will be a prospective, multicenter validation of the proposed templates. Each participating unit will implement the structured format in routine practice, generating data on usability, clarity, and interpretability from both radiologists and referring clinicians. This pilot phase will allow iterative refinement of the tools and establish a platform for outcome-based studies evaluating the real-world impact of structured MRD reporting on diagnostic accuracy, multidisciplinary decision-making, and patient outcomes.

This study is inherently limited by its consensus-based design and the absence of prospective validation, reproducibility analyses, and interobserver agreement assessment. Consequently, the impact of the proposed templates on reporting standardization, diagnostic performance, and clinical decision-making remains to be established. Although the panel was composed exclusively of Italian experts, participants were selected through a purposive sampling strategy based on recognized expertise in pelvic floor disorders and MR defecography. The multidisciplinary panel included highly experienced specialists from high-volume referral centers, providing a robust foundation for the consensus process. Nevertheless, international validation is warranted to confirm the generalizability of the recommendations across different healthcare settings. Finally, implementation feasibility, including workflow integration, reporting time, and user acceptance, was not evaluated and should be addressed in future multicenter studies.

In conclusion, this modified Delphi consensus study established a multidisciplinary framework for standardized MR defecography referral and reporting, integrating a structured clinical referral dataset with a comprehensive reporting template. By involving experts from multiple specialties dedicated to the management of pelvic floor disorders, the study identified a core set of clinically relevant parameters considered essential for the consistent acquisition, interpretation, and communication of MR defecography findings. The proposed framework provides a practical reference for reporting standardization and multidisciplinary communication and may contribute to greater uniformity in MR defecography practice across institutions. Furthermore, it offers a foundation for future research initiatives, educational activities, and the development of shared reporting standards in pelvic floor imaging. Future studies should focus on validating the proposed framework and assessing its reproducibility and applicability in broader clinical settings.
